# Active Observation of Biochemical Recurrence without Treatment following Radical Prostatectomy: Long-Term Analysis of Outcomes

**DOI:** 10.3390/cancers14174078

**Published:** 2022-08-23

**Authors:** Erica Huang, Linda My Huynh, Joshua Tran, Adam M. Gordon, Ryan Chandhoke, Blanca Morales, Douglas Skarecky, Thomas E. Ahlering

**Affiliations:** 1 Department of Urology, University of California, Orange, CA 92868, USA; 2 MD-PhD Scholars Program, University of Nebraska Medical Center, Omaha, NE 69198, USA

**Keywords:** prostate cancer, biochemical recurrence, radiation therapy, androgen deprivation therapy, hormonal therapy, salvage therapy

## Abstract

**Simple Summary:**

This observational study on 407 patients experiencing biochemical recurrence (BCR) following radical prostatectomy (RP) reveals that 33% of men were managed with active observation without risk of prostate-related death (0%), at an average of 7.5 years follow-up. These findings support that a significant portion of men following RP develop a benign recurrence that does not require treatment intervention.

**Abstract:**

Biochemical recurrence (BCR) following radical prostatectomy (RP) is an unreliable predictor of prostate cancer (PC) progression. This study was a retrospective cohort analysis of prospectively collected data (407/1895) of men with BCR at a tertiary referral center. Patients were assessed for active observation (AO) compared with a treatment group (TG) utilizing doubling time (DT) kinetics. Risk assessment was based on the initial DT (>12 vs. <12 months), then based on the DT pattern (changed over time). Those with unstable, rapidly decreasing DTs received treatment. Those with increasing and slowly decreasing DTs prompted observation. The primary outcome was PC mortality, safety, and efficacy of observations based on DT kinetics. The secondary outcome was BCR patients managed with or without treatment. The median follow-up was 7.5 years (IQR 3.9–10.7). The PCSM in TG and AO was 10.7% and 0%, respectively (*p* < 0.001). The initial DT was >12 months in 73.6% of AO versus 22.6% of TG (*p* < 0.001). An increasing DT pattern was observed in 71.5% of AO versus 32.7% of TG (*p* < 0.001). Utilizing the Cleveland Clinic’s PCSM nomogram, at 10 years, predicted and observed PCSM was 8.6% and 9.5% (*p* = 0.78), respectively. In conclusion, one-third of patients with BCR post-RP were managed without treatment using DT kinetics, avoiding treatment-related complications, quality-of-life issues, and expenses.

## 1. Introduction

Generally, prostate cancer recurrence is unlike that of other cancers in that residual disease is dramatically less aggressive and mortal compared with that of most other cancers. In 2018, Bill-Axelson et al. reported the long-term (29 years) mortality of 347 men undergoing radical prostatectomy around 1990 (SPCG-4) [1]: while 81% of men had died at time of the follow-up, only about 5% of GGG 1,2 and 30% of GGG 3, and 50% of GGG 4,5 had died of prostate cancer. The study reinforces that recurrence, even in high-risk men, should be carefully evaluated for risk versus benefit of ADT and other treatments.

Management of prostate cancer (PC) biochemical recurrence (BCR) following radical prostatectomy (RP) remains an important issue [2]. At the time of diagnosis, it is common practice to risk-stratify men to recommend intervention and follow-up [3]. The same is true after RP, when patients are restratified according to the risk of recurrence based on pathological grade or stage and preoperative serum prostate-specific antigen (PSA) concentration [3,4,5]. Much has been published on post-RP surveillance of cancer recurrence using PSA [6,7,8]. While a nondetectable PSA beyond the first 2 years after RP is highly predictive of non-prostate-cancer death [9], biochemical recurrence indicates the need for increased vigilance for problematic cancer recurrence requiring intervention. In addition, men with delayed BCR or BCR with doubling times (DTs) greater than 12 months have a low risk of dying of prostate cancer, with some guidelines recommending observation of these men [10,11].

In 1997, two independent groups: Stamey et al., and deKernion et al., detailed the first in-depth evaluations of postoperative PSA doubling times and their implications for early recurrence and, more importantly, for cancer aggressiveness and clinical progression [12,13]. Following BCR, a PSA DT of less than 6 months is a means to identify patients at high risk of metastatic progression and death from prostate cancer. In 2005, we observed and reported on the oncologic outcomes from the Long Beach Veterans Hospital following RP from 1985 to 2005 based on PSA doubling times of <6, 7–18, and >18 months [14]. Men with DTs longer than 18 months were identified as potential candidates for observation. In 2002, we began carefully following PSA DTs as a potential means to observe men without the need for androgen deprivation treatment (ADT) and or radiation therapy (RT). While we initially assumed that the DT would be reasonably stable, we realized that the DT changed when recalculated after each subsequent PSA. This experience led to the belief that the PSA DT might be a simplified surrogate for PC cell division. We hypothesized that genetically unstable PC cells would result in the hyper-exponential rise in PC cell counts and, thus, PSA levels. As expected, rapidly increasing PSA levels resulted in DTs that decreased over time. We thus engaged an electronic spreadsheet engineer to devise a spreadsheet that recalculated the DT with each new PSA level and date. In 2012, Cary et al. published similar results, noting that DT was not stable based on two PSA DT time points at least 1 year apart. Decreasing DTs suggested that early or immediate treatment intervention should be considered, especially in men with Gleason scores of 8–10 [15].

We also observed a subset of patients whose DT increased over time, suggesting the cells were dividing less frequently. This supports a treatment plan of further observation without secondary treatment. This observational study of untreated men with BCR details their patient demographics, PSA DT kinetics (initial DT and subsequent DT change), and clinical and oncologic outcomes.

## 2. Patients and Methods

Between June 2002 and September 2019, 1865 patients undergoing robot-assisted radical prostatectomy (RARP) by a single surgeon were included in the study. Preoperative demographics, oncologic information, and long-term follow-up were prospectively recorded in an anonymized, electronic database under approved institutional review board protocol at the University of California, Irvine, USA (HS#1998-84). All data collection was conducted in compliance with the Health Insurance Portability and Accountability Act, and federal guidelines for informed consent were followed.

After serial elevation of PSA > 0.1, patients were counseled on possible intervention options according to European Association of Urology (EAU) guidelines [11]. Treatment interventions were guided by previous studies indicating that PSA DT < 12 months and high pathological GGG and stage are associated with a higher risk of cancer progression. Patients classified as EAU low-risk (PSA DT > 12 months) were fully counseled regarding RT and/or ADT and the option of observation [14].

All statistical analyses were based on follow up through 29 March 2021. Patients were initially excluded if they were undergoing cytoreductive (*n* = 3) or simple prostatectomy (*n* = 9), and patients with neuroendocrine/small cell adenocarcinoma (*n* = 3). BCR was defined as two consecutive PSA values of 0.2 ng/mL or adjuvant therapy (*n* = 53). Amongst 407 patients who experienced BCR, 162 patients did not have sufficient numbers of PSAs to calculate PSADT before undergoing adjuvant or salvage therapy. A total of 245 patients had enough PSAs to calculate an initial DT calculated based on the first 3–4 PSAs after BCR (0.2 ng/mL, ×2). A PSA DT calculator and tracker was designed, using the formula ln(2)/log (slope of linear regression line of log PSA vs. time) [16]. The PSA doubling time was recalculated and tracked with each new PSA value referenced to the date of surgery. DT pattern (increasing or decreasing) was assessed based on the change in DT over time.

A total of 271 patients underwent intervention including RT and ADT (*n* = 115) or ADT alone (*n* = 156). Of the 271 patients, the 162 who had a rapidly rising PSA or extensive local metastatic disease were counseled and immediately treated with RT and ADT or ADT alone following treatment counseling at our center or their local center. A total of 109 patients progressed after the PSADT was calculated and then counseled (Tumor Board) for treatment options; 136 patients were not treated and instead underwent active observation (AO) with close monitoring of PSA and DT.

## 3. Statistical Methods and Analysis

To evaluate demographic differences between observation and treatment groups (TG), descriptive statistics were conducted via Student’s *t*-test for continuous variables and test of proportions or ANOVA for categorical variables. We conducted 15-year Kaplan–Meier survival analyses between observation and treatment groups to evaluate overall survival (OS) and prostate-cancer-specific survival (PCSS). Patients were censored at death or the last follow-up.

To assess our study group’s prostate-cancer-specific mortality outcomes compared with United States PC mortality outcomes, we performed a noninferiority PCSM risk analysis on our patients using the Cleveland Clinic’s PCSM risk calculator, and observed and predicted PCSMs were compared via chi-squared analysis [17].

All statistical tests and figures were conducted and produced in R statistical package (R Foundation for Statistical Computing, Vienna, Austria).

## 4. Results

Table 1 displays the demographic information based on treatment intervention or observation with no difference in follow-up. However, there were expected significant differences in oncologic characteristics: preoperative PSA, postoperative PSAdt, pGGG, *p*-stage, and positive margin rates. There were substantial differences between groups for PCSM (AO: 0%, TG: 10.7%) and OM (AO: 9.6%, TG: 18.5%).

Table 2 depicts the detailed demographics of the AO group stratified by pathological GGG. GGGs 2 and 3 comprised 67.9% of the patients, and GGGs 4 and 5 comprised 20.6%, compared with 12.5% in GGG 1. As previously reported, DT is the most important prognostic metric once BCR has occurred. The proportion of men with an initial DT > 12 months dropped from 94.1% in GGG 1, progressively reducing as GGGs increased, to 57.1% in GGG 4,5. Furthermore, the percentage of men with initial DTs of 6–12 months progressively to 28.6% in GGG 4,5. Initial DTs < 6 months occurred only in GGG 4,5.

Once an initial DT was established, the DT pattern was repeatedly assessed over time as stable, decreasing, or increasing. Men with DT < 12 months and decreasing DT were considered high risk and counseled for treatment. However, men with a very slowly decreasing DT (typically elderly men) were counseled about treatment and further observation. Importantly, the AO group had 68% with increasing DT, but 27% had decreasing DT patterns (Table 1).

In the 15-year Kaplan–Meier analysis, the AO group with no RT/ADT treatment had 100% PCSS (*p* < 0.001) and had better (trending) OS than the treated group (*p* = 0.092).

We performed a noninferiority analysis via Cleveland Clinic’s nomogram for PCSM risk [17]. Predicted versus observed PCSM of at 5 years was 3.8% compared with 3.1%, respectively (*p* = 0.64). At 10 years, predicted and observed PCSM was 8.6% and 9.5% (*p* = 0.78), respectively (Supplementary Table S1).

## 5. Discussion

DT after RP is recognized as the most accurate harbinger of metastasis and prostate cancer death [7,9,10]. Patel and Pound commented on men with longer DTs, defined as >12–15 months, indicating that they were at a “reduced” risk of metastasis and death [7,13]. In a similar fashion, we and others have reported that men with DTs > 12–18 months are at “lower risk” of metastatic progression [10,14]. Although these men have been recognized to be at reduced risk of progression and death, there has been no in-depth portrayal of how they fare. Additionally, if or when salvage treatment should be initiated has never been well-characterized. Because BCR is an imprecise predictor of progression and PC death, it is also logical to see if men can be risk-stratified for treatment (none or delayed) using DT to avoid treatment-related complications, reduction in quality of life, and expense.

The most important finding of this study is that one-third of the BCRs were safely managed using PSA DT without RT, RT/ADT, or ADT treatment. There was no significant difference in age or BMI between AO patients and men undergoing treatment (Table 1). Table 2 notes the important demographics of the AO group, broken down by GGG.

**Figure 1:**
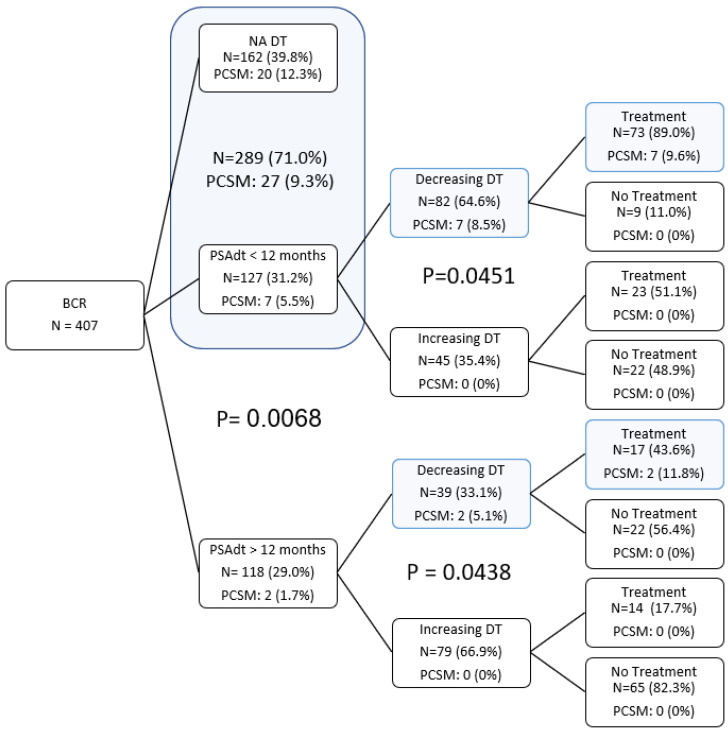
Decision trees: Initial separation point was as PSADT greater or less than 12 months. The second division point is based on the pattern of how the PSADT changed (increasing versus decreasing) over time. *p*-values are for PCSM via chi-squared analysis.

Figure 1 displays PCSM according to initial DT and subsequent DT pattern. Those with adverse PSA progression who were treated prior to DT determination (NA DT) had a PCSM of 12.3%. In a similar fashion, PCSM was 8.5% in those with a calculable initial DT < 12 months and a decreasing DT pattern. We also cautioned regarding those with DT > 12 months and decreasing DT pattern, with PCSM of 5.1%. While initial DT is important in guiding treatment intervention, Figure 1 showcases the necessity and usefulness of observing how DT pattern changes over time. However, those with increasing DT patterns had no PCSM to date, regardless of initial DT.

Seventy-three men (89%) with DT < 12 months had a DT pattern that decreased over time and were managed with salvage intervention. PCSM was 9.6%, comparable to that of the NA DT group treated prior to established DT. We were cautious with those untreated in this group who were being strongly considered for treatment. However, 48 (35%) with an initial DT < 12 months had an increasing DT and 0% PCSM. The initial DT, but more importantly, the DT change over time, helped assess the need for treatment. For men with an initial DT > 12 months and an increasing DT pattern, 82% (65 men) did not receive ADT or RT/ADT intervention. The other 18% (14 men) were primarily treated due to concern for young age. For example, a 55-year-old with a predicted PSA above 50 ng/mL in twenty years would be a strong candidate for treatment.

In men with initial DT > 12 months and decreasing DT, 53% (27 men) did not receive treatment, and were typically characterized as over 70 years of age, initial DT > 36 months, and slowly decreasing DT patterns. We continued to observe these patients unless there was a rapid progression of the PSA.

After the prostate is removed, rising PSA values are theoretically a surrogate for tumor growth, as PSA elevation is presumed to reflect residual prostate cancer cells. However, it is also possible that the PSA concentrations rise due to residual benign prostate cells, possibly in the bladder neck or urethra. It would follow that persistent cancer cells grow in a stable exponential fashion, while more aggressive cells may be more genetically degenerate. In this case, modeling would predict that cells more rapidly divide, and the DT should decrease over time. Similarly, if the DT is increasing, we can hypothesize that the cells are taking longer intervals to divide. We suggest that an increasing DT pattern represents “benign” cells that are not progressing in a malignant manner. These cells would not be expected to produce a problematic exponential PSA rise but rather a more linear one, as seen in men with benign prostatic hyperplasia [15]. In some unique scenarios, DT can increase or decrease due to other reasons. We observed that DT can increase as a consequence of improved diet and exercise and decrease due to weight gain, metabolic syndrome, and stress.

**Figure 2:**
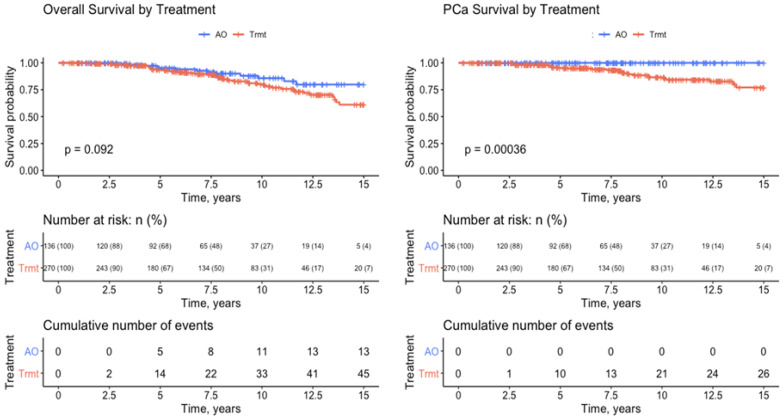
A 15-year Kaplan–Meier analysis of overall survival and prostate-cancer-specific survival.

Looking ahead, it is important to envision clinical trials that do not over- or undertreat men following RP. Our data suggest the key to safely observing these patients is the ability to monitor PSA doubling time (Figure 2). To assess the safety and efficacy of treating patients with AO, we conducted a noninferiority survival analysis using the validated Cleveland Clinic PCSM nomogram by Brockman et al. [17]. Our patients had comparable predicted and expected PCSM at 5 and 10 years post-RP, demonstrating noninferiority despite more conservative treatment of BCR patients.

Thus, if DTs are long and predictably increasing, patients can be managed with observation without intervention. Furthermore, if the DT is short and following a decreasing pattern (Figure 2), intervention (hormonal, radiation therapy, etc.) should be immediately discussed and pursued. However, we note there were 2/118 (1.8%) PCSM amongst men with initial DT > 12 months (Figure 1). One patient remained undetectable for 19 months post-RP (pre-op PSA 5.9, Gleason 3 + 4, pT2B, 72.7 years old at RP). Even though he was relatively low risk, he experienced progression on ADT treatment and died 8 years post-RP (age 80.7 years). The other patient was undetectable for 47 months post-RP (pre-op PSA 6.6, Gleason 4 + 3, pT3a + 3b, 70.6 years old at RP). Six years post-RP, the patient experienced severe depression that may have influenced his rapid progression and died 8 years post-RP (age 78.7 years). Nevertheless, this is the first paper suggesting that, in general, as long as DT is not decreasing, it appears reasonable to continue monitoring PSA, avoiding the burden of overtreatment.

The key benefit of utilizing DT kinetics is to avoid overtreatment. The side effects of castration have an obvious negative quality of life consequences as well as increased cardiovascular morbidity and mortality [18]. Similar side effects are true with radiation, in addition to a 5% increased rectal and bladder cancer risk [19,20,21,22,23]. There are significant monetary expenses associated with radiation and ADT treatment. For example, the expense of radiation accounting for rectal bleeding and urinary toxicities is approximately USD 26,343 [24,25].

The primary weakness of this study is the retrospective analysis of prospectively collected data. This study could not have been conducted as a randomized trial as the concept of treatment based on DT had not been previously studied. Because the data were collected without a significant understanding of DT kinetics, there was little bias in the prospective collection of data. Another weakness is the average follow-up of 7.5 years, which may not be adequate given the long course of prostate cancer. We note that 25% (*n* = 34) of men in the AO group had a follow-up greater than 10 years with stable increasing DT.

PSA kinetics can logically be used to predict “benign” clinical outcomes in this subset of patients who have BCR. Furthermore, with the growing interest in the use of PSMA PET/CT scans to guide salvage procedures, we foresee this new technology as an important adjunct. PSMA PET/CT scans were introduced beginning in 2017–2018 in the United States. PSMA PET/CT scan findings have begun to be utilized in the management of men with BCR, which will only increase as time progresses. In the future, we envision that salvage procedures (radiation or surgical) of recurrent disease will also guide management of these men before and after their salvage procedures based on how their PSADT kinetics change. We envision that future studies validating DT kinetics to predict treatment necessity may be used in conjunction with machine learning to produce models capable of assisting physicians and patients in accurately determining the need for treatment courses.

## 6. Conclusions

The findings of this observational study of 407 men with BCR after RP support that a substantial proportion can be managed with active observation. They further suggest that age, initial DT, and subsequent patterns of DT change can be used to safely observe patients with little or no apparent risk of PCSM. The men who experience a “benign” recurrence cannot be predicted with standard pathologic parameters. Although many men with BCR require intervention, there exists a distinct subset (33% in this study) who can be directed by DT such that treatment can be avoided or delayed, reducing complications and costs of overtreatment.

## Figures and Tables

**Table 1:** Demographics of all 407 BCR patients: 136 AO and 271 treated (TG) patients.

Treatment	No Trmt	Trmt	Total	
	**Count (%)**	**Count (%)**	**Count (%)**
*n*, all patients	136 (33.4%)	271 (66.6%)	407 (100%)
	**Mean (SD)**	**Mean (SD)**	**Mean (SD)**	***p*-value**
Age, years	63.5 (7.3)	63.8 (7.2)	63.7 (7.3)	0.677
Adj Pre-PSA, ng/mL	8.4 (5.7)	12.6 (16.9)	11.2 (14.3)	0.005
SHIM	19.8 (7.1)	17.9 (7.5)	18.6 (7.4)	0.023
EBL	102.2 (48.4)	96.2 (37.7)	98.2 (41.7)	0.171
BMI	27.0 (3.8)	27.3 (3.8)	27.2 (3.8)	0.467
Prostate Weight	51.4 (21.3)	53.5 (19.4)	52.8 (20.1)	0.337
Follow Up, years	7.5 (4.0)	7.7 (4.4)	7.6 (4.3)	0.688
Time to Death, years	6.9 (2.7)	7.8 (4.0)	7.6 (3.8)	0.426
Time to Earliest Treatment	NA	3.0 (7.7)	3.0 (7.7)	
Current PSADT, months	26.0 (19.9)	8.5 (9.1)	15.6 (16.9)	<0.001
PSADT after 0.2, months	39.4 (294.9)	12.6 (48.4)	23.6 (192.6)	0.272
	**Count (%)**	**Count (%)**	**Count (%)**	***p*-value**
Positive Margins	36 (26.5%)	109 (40.2%)	145 (35.6%)	0.006
*p*-stage				<0.001
pT2	67 (49.3%)	70 (25.8%)	137 (33.7%)	
pT3/T4	69 (50.7%)	201 (74.2%)	270 (66.3%)	
Gleason Grade Group (GGG)				<0.001
1	17 (12.5%)	4 (1.5%)	21 (5.2%)	
2	48 (35.3%)	52 (19.2%)	100 (24.6%)	
3	43 (31.6%)	79 (29.2%)	122 (30.0%)	
4	17 (12.5%)	22 (8.1%)	39 (9.6%)	
5	11 (8.1%)	114 (42.1%)	125 (30.7%)	
PSADT > 0.2 Group, months				<0.001
>12	90 (73.8%)	37 (22.6%)	127 (44.4%)	
6 to 12	19 (15.6%)	48 (29.3%)	67 (23.4%)	
<6	13 (10.7%)	79 (48.2%)	92 (32.2%)	
NA	14 *	107 **	121	
DT Pattern				<0.001
Increasing	96 (72.7%)	49 (32.7%)	142 (50.7%)	
Decreasing	36 (27.3%)	101 (67.3%)	138 (49.3%)	
NA	4 ***	121 **	127	
PCSM	0 (0.0%)	29 (10.7%)	29 (7.1%)	<0.001
Dead	13 (9.6%)	50 (18.5%)	63 (15.5%)	0.019

* Not enough PSAs prior to non-cancer-specific death (*n* = 2), not enough PSAs post-BCR to establish PSA (*n* = 12); ** No PSADT as treatment was initiated based on very rapid PSA progression; *** Not enough PSAs prior to non-cancer-specific death (*n* = 2), lost to follow-up (*n* = 1), and after BCR (*n* = 1). AO = Active Observation; Trmt = Treatment; SD = Standard Deviation; PSA = Prostate-Specific Antigen; SHIM = Sexual Health Inventory for Men; EBL = Estimated Blood Loss; BMI = Body Mass Index; PSADT = PSA Doubling Time Pattern; *p-*stage = Pathological Stage; NA = Not Available; PCSM = Prostate Cancer Specific Mortality.

**Table 2:** Demographics of active observation group by GGG.

Gleason Grade Group, AS	1	2	3	4 and 5	Total	
	**Count (%)**	**Count (%)**	**Count (%)**	**Count (%)**	**Count (%)**
*n*, all patients	17 (12.5%)	48 (35.3%)	43 (31.6%)	28 (20.6%)	136 (100%)
	**Mean (SD)**	**Mean (SD)**	**Mean (SD)**	**Mean (SD)**	**Mean (SD)**	***p*-value**
Age, years	61.2 (7.2)	62.0 (7.2)	63.7 (7.6)	67.0 (6.1)	63.5 (7.3)	0.014
Adj Pre-PSA, ng/mL	6.4 (3.1)	9.6 (7.1)	7.4 (4.9)	9.0 (5.1)	8.4 (5.7)	0.121
SHIM	20.0 (7.2)	21.1 (5.1)	19.9 (8.2)	17.2 (8.2)	19.8 (7.1)	0.169
EBL	113.2 (41.6)	112.5 (61.7)	93.0 (41.3)	92.0 (30.5)	102.2 (48.4)	0.118
BMI	28.5 (4.6)	26.5 (3.5)	26.8 (3.8)	27.3 (3.9)	27.0 (3.8)	0.348
Prostate Weight	54.3 (23.8)	48.4 (16.0)	53.7 (24.3)	51.7 (23.6)	51.4 (21.3)	0.644
Follow Up, years	9.9 (3.3)	8.2 (4.2)	7.1 (3.4)	5.4 (4.2)	7.5 (4.0)	0.001
Time to Death, years	8.6 (2.3)	5.2 (2.3)	6.8 (3.0)	5.2 (3.0)	6.9 (2.7)	0.417
Current PSAdt, months	37.8 (18.4)	28.5 (18.2)	24.0 (20.4)	18.9 (20.3)	26.0 (19.9)	0.02
	**Count (%)**	**Count (%)**	**Count (%)**	**Count (%)**	**Count (%)**	***p*-value**
Positive Margins	3 (17.6%)	12 (25.0%)	14 (32.6%)	7 (25.0%)	36 (26.5%)	0.663
*p*-stage						0.004
pT2	15 (88.2%)	24 (50.0%)	18 (41.9%)	10 (35.7%)	67 (49.3%)	
pT3/T4	2 (11.8%)	24 (50.0%)	25 (58.1%)	18 (64.3%)	69 (50.7%)	
Initial PSAdt Group, months						0.005
>12	16 (94.1%)	42 (87.5%)	34 (79.1%)	16 (57.1%)	108 (79.4%)	
6 to 12	1 (5.9%)	5 (10.4%)	9 (20.9%)	8 (28.6%)	23 (16.9%)	
<6	0 (0.0%)	1 (2.1%)	0 (0.0%)	4 (14.3%)	5 (3.7%)	
DT Pattern						0.488
Increasing	12 (75.0%)	38 (82.6%)	28 (65.1%)	18 (66.7%)	96 (72.7%)	
Decreasing	4 (25.0%)	8 (17.4%)	15 (34.9%)	9 (33.3%)	36 (27.3%)	
NA *	1	2	0	1	4	
PCSM	0 (0.0%)	0 (0.0%)	0 (0.0%)	0 (0.0%)	0 (0.0%)	NA
Dead	4 (23.5%)	2 (4.2%)	5 (11.6%)	2 (7.1%)	13 (9.6%)	0.119

* NA DT pattern (*n* = 4): not enough PSAs prior to non-cancer-specific death (*n* = 2), lost to follow-up (*n* = 1), and after BCR (*n* = 1). AO = Active Observation; SD = Standard Deviation; PSA = Prostate-Specific Antigen; SHIM = Sexual Health Inventory for Men; EBL = Estimated Blood Loss; BMI = Body Mass Index; PSADT = PSA Doubling Time Pattern; *p-*stage = Pathological Stage; NA = Not Available; PCSM = Prostate Cancer Specific Mortality.

## Data Availability

The data presented in this study are available on request from the corresponding author.

## Supplementary Materials

The following supporting information can be downloaded at: https://www.mdpi.com/article/10.3390/cancers14174078/s1, Table S1: Noninferiority analysis of observed versus predicted PCSM.

## References

[1] Bill-Axelson A., Holmberg L., Garmo H., Taari K., Busch C., Nordling S., Häggman M., Andersson S.-O., Andrén O., Steineck G. (2018). Radical Prostatectomy or Watchful Waiting in Prostate Cancer—29-Year Follow-Up. N. Engl. J. Med..

[2] D’Amico A.V., Whittington R., Malkowicz S.B., Schultz D., Blank K., Broderick G.A., Tomaszewski J.E., Renshaw A.A., Kaplan I., Beard C.J. (1998). Biochemical Outcome after Radical Prostatectomy, External Beam Radiation Therapy, or Interstitial Radiation Therapy for Clinically Localized Prostate Cancer. JAMA.

[3] Partin A.W., Kattan M.W., Subong E.N., Walsh P.C., Wojno K.J., Oesterling J.E., Scardino P.T., Pearson J.D. (1997). Combination of Prostate-Specific Antigen, Clinical Stage, and Gleason Score to Predict Pathological Stage of Localized Prostate Cancer. A Multi-Institutional Update. JAMA.

[4] Kattan M.W., Eastham J.A., Stapleton A.M., Wheeler T.M., Scardino P.T. (1998). A Preoperative Nomogram for Disease Recurrence Following Radical Prostatectomy for Prostate Cancer. J. Natl. Cancer Inst..

[5] Cooperberg M.R., Pasta D.J., Elkin E.P., Litwin M.S., Latini D.M., Du Chane J., Carroll P.R. (2005). The University of California, San Francisco Cancer of the Prostate Risk Assessment Score: A Straightforward and Reliable Preoperative Predictor of Disease Recurrence after Radical Prostatectomy. J. Urol..

[6] Trapasso J.G., deKernion J.B., Smith R.B., Dorey F. (1994). The Incidence and Significance of Detectable Levels of Serum Prostate Specific Antigen after Radical Prostatectomy. J. Urol..

[7] Pound C.R., Partin A.W., Eisenberger M.A., Chan D.W., Pearson J.D., Walsh P.C. (1999). Natural History of Progression after PSA Elevation Following Radical Prostatectomy. JAMA.

[8] Kabalin J.N., McNeal J.E., Johnstone I.M., Stamey T.A. (1995). Serum Prostate-Specific Antigen and the Biologic Progression of Prostate Cancer. Urology.

[9] Boorjian S.A., Thompson R.H., Tollefson M.K., Rangel L.J., Bergstralh E.J., Blute M.L., Karnes R.J. (2011). Long-Term Risk of Clinical Progression after Biochemical Recurrence Following Radical Prostatectomy: The Impact of Time from Surgery to Recurrence. Eur. Urol..

[10] Freedland S.J., Humphreys E.B., Mangold L.A., Eisenberger M., Dorey F.J., Walsh P.C., Partin A.W. (2005). Risk of Prostate Cancer-Specific Mortality Following Biochemical Recurrence after Radical Prostatectomy. JAMA.

[11] Cornford P., van den Bergh R.C.N., Briers E., Van den Broeck T., Cumberbatch M.G., De Santis M., Fanti S., Fossati N., Gandaglia G., Gillessen S. (2021). EAU-EANM-ESTRO-ESUR-SIOG Guidelines on Prostate Cancer. Part II-2020 Update: Treatment of Relapsing and Metastatic Prostate Cancer. Eur. Urol..

[12] Pruthi R.S., Johnstone I., Tu I.P., Stamey T.A. (1997). Prostate-Specific Antigen Doubling Times in Patients Who Have Failed Radical Prostatectomy: Correlation with Histologic Characteristics of the Primary Cancer. Urology.

[13] Patel A., Dorey F., Franklin J., deKernion J.B. (1997). Recurrence Patterns after Radical Retropubic Prostatectomy: Clinical Usefulness of Prostate Specific Antigen Doubling Times and Log Slope Prostate Specific Antigen. J. Urol..

[14] Ahlering T.E., Skarecky D.W. (2005). Long-Term Outcome of Detectable PSA Levels after Radical Prostatectomy. Prostate Cancer Prostatic Dis..

[15] Cary K.C., Johnson C.S., Cheng L., Koch M.O. (2012). A Critical Assessment of Post-Prostatectomy Prostate Specific Antigen Doubling Time Acceleration—Is It Stable?. J. Urol..

[16] Prostate Cancer Nomograms: PSA Doubling Time|Memorial Sloan Kettering Cancer Center. https://www.mskcc.org/nomograms/prostate/psa_doubling_time.

[17] Brockman J.A., Alanee S., Vickers A.J., Scardino P.T., Wood D.P., Kibel A.S., Lin D.W., Bianco F.J., Rabah D.M., Klein E.A. (2015). Nomogram Predicting Prostate Cancer-Specific Mortality for Men with Biochemical Recurrence After Radical Prostatectomy. Eur. Urol..

[18] Tucci M., Leone G., Buttigliero C., Zichi C., DI Stefano R.F., Pignataro D., Vignani F., Scagliotti G.V., DI Maio M. (2018). Hormonal Treatment and Quality of Life of Prostate Cancer Patients: New Evidence. Minerva Urol. E Nefrol. Ital. J. Urol. Nephrol..

[19] Baxter N.N., Tepper J.E., Durham S.B., Rothenberger D.A., Virnig B.A. (2005). Increased Risk of Rectal Cancer after Prostate Radiation: A Population-Based Study. Gastroenterology.

[20] Nieder A.M., Porter M.P., Soloway M.S. (2008). Radiation Therapy for Prostate Cancer Increases Subsequent Risk of Bladder and Rectal Cancer: A Population Based Cohort Study. J. Urol..

[21] Sountoulides P., Koletsas N., Kikidakis D., Paschalidis K., Sofikitis N. (2010). Secondary Malignancies Following Radiotherapy for Prostate Cancer. Ther. Adv. Urol..

[22] Moon K., Stukenborg G.J., Keim J., Theodorescu D. (2006). Cancer Incidence after Localized Therapy for Prostate Cancer. Cancer.

[23] Parker C.C., Clarke N.W., Cook A.D., Kynaston H.G., Petersen P.M., Catton C., Cross W., Logue J., Parulekar W., Payne H. (2020). Timing of Radiotherapy after Radical Prostatectomy (RADICALS-RT): A Randomised, Controlled Phase 3 Trial. Lancet.

[24] Su H.-W. (2019). Comparison of Oncological Outcomes and Cost of Adjuvant Radiation versus Observation for Post-Radical Prostatectomy Patients.

[25] Showalter T.N., Foley K.A., Jutkowitz E., Lallas C.D., Trabulsi E.J., Gomella L.G., Dicker A.P., Pizzi L.T. (2012). Costs of Early Adjuvant Radiation Therapy after Radical Prostatectomy: A Decision Analysis. Ann. Oncol. Off. J. Eur. Soc. Med. Oncol..

