# Supplementary material for: Active Observation of Biochemical Recurrence without Treatment following Radical Prostatectomy: Long-Term Analysis of Outcomes
Source: Cancers (Basel). 2022 Aug 23;14(17):4078. doi: 10.3390/cancers14174078 (PMC9454648; doi:10.3390/cancers14174078)
Supplement: Supplementary file 1 [file cancers-14-04078-s001.zip › cancers-1848878-supplementary.pdf]

Table S1. Noninferiority analysis of observed versus predicted PCSM.

Time (Years)	Our PCSM	*n*	Predicted PCSM (Brockman et al.)	*n*	*p*-value
5	0.031	277	0.0383	327	0.6402
10	0.095	136	0.086	327	0.7836

PCSM = Prostate Cancer Specific Mortality
